# Study design considerations for strategies in early interventions in Alzheimer’s disease

**DOI:** 10.1002/alz.094707

**Published:** 2025-01-09

**Authors:** Samuel P. Dickson

**Affiliations:** ^1^ Pentara Corporation, Salt Lake City, UT USA

## Abstract

**Background:**

Alzheimer’s disease (AD) treatments targeting disease modification face a development paradox: the cascade of events that lead to AD may already have irreversible consequences at diagnosis, so the most treatable patients won’t progress enough to show significance at a sample size or timeframe that most sponsors can afford. Though monoclonal antibodies (mABs) have seen some recent success, the limitations of mABs have led many to consider designs that either build on the success of mABs, or start earlier in disease progression by pursuing secondary prevention for subclinical patients, or primary prevention. We present statistical considerations for these designs with enrichment strategies.

**Method:**

We use AD risk by age and ApoE genotype and other historic data to estimate event rates and sample sizes for primary and secondary prevention studies and for studies of combination treatment with mABs. We consider both event‐based and continuous‐endpoint analyses for biomarkers or clinical outcomes. We assume a 30% reduction in events for treatment. The primary population targets ages 65 to 85, but can be enriched by targeting later ages or alternative demographics like the Down syndrome (DS) population.

**Result:**

A 1.3% base event rate is anticipated in primary prevention studies. For 80% power this may require more than 20,000 participants over 5 years and close to 30,000 when accounting for 30% dropout. Enrichment by raising the minimum age by 10 years or including only ApoE4 carriers or participants with DS reduces sample size requirements by half. Using a continuous biomarker outcome will also reduce sample size to approximately 10,000 to 15,000 participants. Secondary prevention study sample sizes may be reduced to 1,000 to 6,000 subjects over a two‐year treatment period, while combination studies may have 500 to 2,000 subjects. A digital assessment that reduced variability 30% would reduce sample size by half.

**Conclusion:**

Early intervention and combination treatment studies may require larger sample sizes than have been used in most AD clinical trials, but the recent successes of mABs and the potential gains in safety and efficacy may justify the additional resources.

